# 2-[2-(2,6-Di­chloro­benz­yloxy)-2-phenyl­eth­yl]-2*H*-indazole

**DOI:** 10.1107/S1600536814004887

**Published:** 2014-03-08

**Authors:** Özden Özel Güven, Gökhan Türk, Philip D. F. Adler, Simon J. Coles, Tuncer Hökelek

**Affiliations:** a Department of Chemistry, Bülent Ecevit University, 67100 Zonguldak, Turkey; b Department of Chemistry, Southampton University, SO17 1BJ Southampton, England; c Department of Physics, Hacettepe University, 06800 Beytepe, Ankara, Turkey

## Abstract

In the title compound, C_22_H_18_Cl_2_N_2_O, the indazole ring system is approximately planar [maximum deviation = 0.031 (2) Å], its mean plane is oriented at 3.17 (4) and 19.34 (4)° with respect to the phenyl and benzene rings. In the crystal, weak C—H⋯π inter­actions link the mol­ecules into supra­molecular chains running along the *b*-axis direction.

## Related literature   

For clinical uses of azole anti­fungals possessing an imidazole ring such as micozanole and econazole, see: Godefroi *et al.* (1969 ▶). Some indazole derivatives have been known as antifungal also, see: Lebouvier *et al.* (2007 ▶); Park *et al.* (2007 ▶). For related structures, see: Freer *et al.* (1986 ▶); Özel Güven *et al.* (2008 ▶, 2010 ▶, 2013 ▶); Peeters *et al.* (1979 ▶).
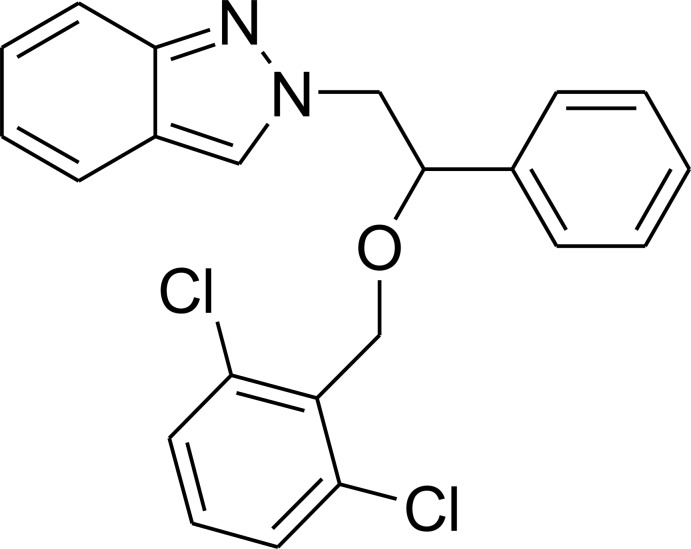


## Experimental   

### 

#### Crystal data  

C_22_H_18_Cl_2_N_2_O
*M* *_r_* = 397.28
Monoclinic, 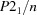

*a* = 15.2399 (4) Å
*b* = 5.3814 (3) Å
*c* = 23.0461 (6) Å
β = 90.871 (3)°
*V* = 1889.84 (13) Å^3^
*Z* = 4
Mo *K*α radiation
μ = 0.36 mm^−1^
*T* = 294 K
0.35 × 0.20 × 0.15 mm

#### Data collection  

Rigaku Saturn724+ diffractometer
Absorption correction: multi-scan (*CrystalClear-SM Expert*; Rigaku, 2011 ▶) *T*_min_ = 0.918, *T*_max_ = 0.948
18118 measured reflections
4737 independent reflections
3685 reflections with *I* > 2σ(*I*)
*R*_int_ = 0.038
3 standard reflections every 120 min intensity decay: 1%

#### Refinement  

*R*[*F* ^2^ > 2σ(*F* ^2^)] = 0.043
*wR*(*F* ^2^) = 0.109
*S* = 1.08
4737 reflections
248 parameters
H atoms treated by a mixture of independent and constrained refinement
Δρ_max_ = 0.90 e Å^−3^
Δρ_min_ = −0.39 e Å^−3^

### 

Data collection: *CrystalClear-SM Expert* (Rigaku, 2011 ▶); cell refinement: *CrystalClear-SM Expert*; data reduction: *CrystalClear-SM Expert*; program(s) used to solve structure: *SHELXS97* (Sheldrick, 2008 ▶); program(s) used to refine structure: *SHELXL97* (Sheldrick, 2008 ▶); molecular graphics: *ORTEP-3 for Windows* (Farrugia, 2012 ▶); software used to prepare material for publication: *WinGX* (Farrugia, 2012 ▶) and *PLATON* (Spek, 2009 ▶).

## Supplementary Material

Crystal structure: contains datablock(s) I, global. DOI: 10.1107/S1600536814004887/xu5774sup1.cif

Structure factors: contains datablock(s) I. DOI: 10.1107/S1600536814004887/xu5774Isup2.hkl

Supporting information file. DOI: 10.1107/S1600536814004887/xu5774Isup3.cml

CCDC reference: 989513

Additional supporting information:  crystallographic information; 3D view; checkCIF report

## Figures and Tables

**Table 1:** Hydrogen-bond geometry (Å, °) *Cg*2 and *Cg*3 are the centroids of the C2–C7 and C10–C15 rings, respectively.

*D*—H⋯*A*	*D*—H	H⋯*A*	*D*⋯*A*	*D*—H⋯*A*
C4—H4⋯*Cg*3^i^	0.93	2.91	3.591 (2)	131
C11—H11⋯*Cg*2^i^	0.93	2.87	3.616 (2)	138

Symmetry code: (i) 
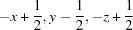
.

**Fig. 1.**
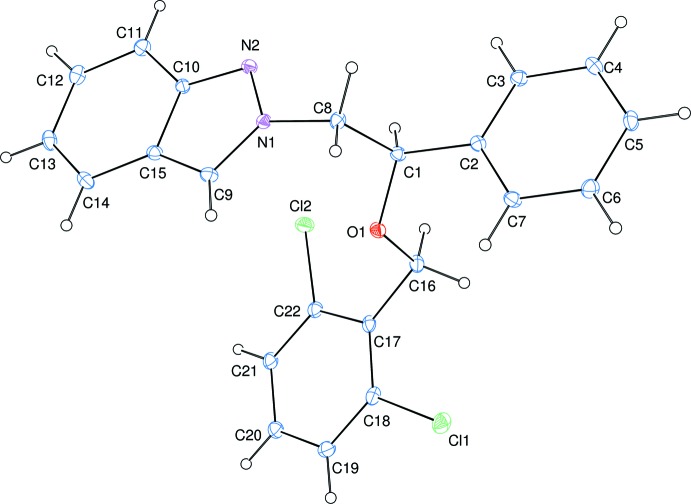
The molecular structure of the title compound with the atom-numbering scheme. Displacement ellipsoids are drawn at the 50% probability level.

**Fig. 2.**
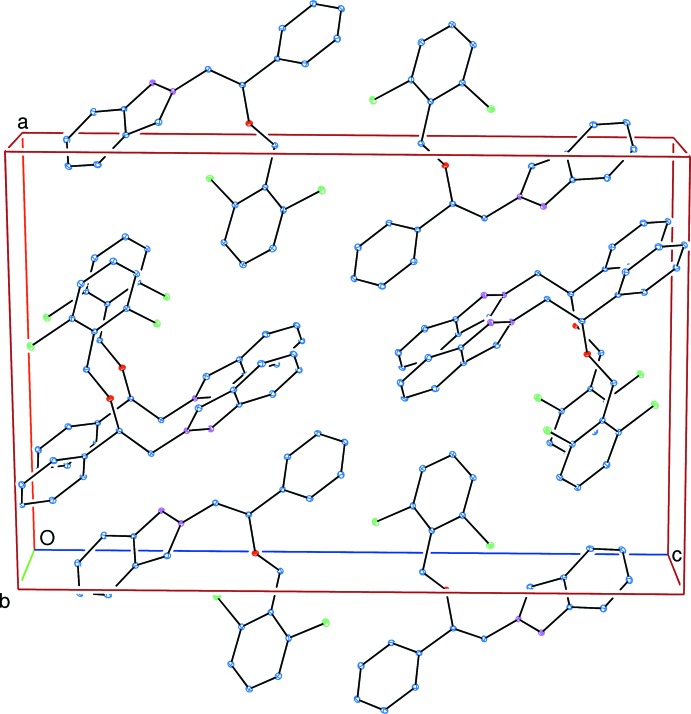
A view of the crystal packing of the title compound [H-atoms have been omitted for clarity].

## supplementary crystallographic information

### 1. Comment

The azole antifungals possesing an imidazole ring such as miconazole and econazole have been developed for clinical uses (Godefroi *et al.*, 1969). Some indazole derivatives have been known as antifungal also (Lebouvier *et al.*, 2007; Park *et al.*, 2007). The crystal structure of indazole group containing ketone has been reported recently (Özel Güven *et al.*, 2013). The crystal structures of imidazole ring containg ethers like miconazole (Peeters *et al.*, 1979) and econazole (Freer *et al.*, 1986) have been reported before. The crystal structures of benzimidazole ring containing ether (Özel Güven *et al.*, 2008) and 1,2,4-triazole ring containg ether have been reported previously (Özel Güven *et al.*, 2010). Now, we report herein the crystal structure of the title indazole derivative, (I).

In the molecule of the title compound (Fig. 1), the bond lengths and angles are generally within normal ranges. The indazole [*B* (N1/N2/(C9—C15)] ring system is approximately planar with a maximum deviation of -0.031 (2)Å (for atom C12). Its mean plane is oriented with respect to the phenyl [*A* (C2—C7)] and benzene [ *C* (C17—C22)] rings at dihedral angles of A/B = 3.17 (4) and B/C = 19.34 (4) °. The dihedral angle between phenyl and benzene rings is A/C = 17.20 (5)°. Atom C8 is 0.016 (2) Å away from the indazole ring plane, while atoms C1 and O1 are -0.026 (2) and 0.599 (1) Å away from the phenyl ring plane. On the other hand, atoms Cl1, Cl2 and C16 are at distances of -0.0258 (5), -0.0693 (5) and -0.074 (2) Å to the benzene ring plane.

In the crystal structure, weak C—H···π interactions (Table 1) may be effective in the stabilization of the structure.

### 2. Experimental

The title compound, (I), was synthesized by the reaction of 1-phenyl-2-(2*H*-indazol-2-yl)ethanol with NaH and appropriate benzyl halide. NaH (0.025 g, 0.63 mmol) was added in small fractions to a solution of alcohol (0.150 g, 0.63 mmol) in DMF (3-4 ml). Then, appropriate benzyl halide (0.151 g, 0.63 mmol) was added dropwise. The mixture was stirred at room temperature for 3 h, and the excess hydride was decomposed with a small amount of methyl alcohol. After evaporation to dryness under reduced pressure, small amount of water was added and extracted with methylene chloride. The organic layer was separated, dried over anhydrous sodium sulfate, and then evaporated to dryness. The crude residue was purified by chromatography on a silica-gel column using hexane-ethyl acetate mixture (10:1) as eluent. The ether was recrystallized from 2-propanol to obtain colourless crystals suitable for X-ray analysis (yield; 0.178 g, 71%).

### 3. Refinement

Atom H9 (for C9) was located in a difference Fourier map and was refined freely. The remaining H atoms were positioned geometrically with C—H = 0.93, 0.97 and 0.98 Å for aromatic, methylene and methine H, respectively, and constrained to ride on their parent atoms, with *U*_iso_(H) = 1.2 *U*_eq_(C).

### Crystal data

**Table:**

C_22_H_18_Cl_2_N_2_O	*F*(000) = 824
*M**_r_* = 397.28	*D*_x_ = 1.396 Mg m^−^^3^
Monoclinic, *P*2_1_/*n*	Mo *K*α radiation, λ = 0.71073 Å
Hall symbol: -P 2yn	Cell parameters from 14300 reflections
*a* = 15.2399 (4) Å	θ = 3.2–28.7°
*b* = 5.3814 (3) Å	µ = 0.36 mm^−^^1^
*c* = 23.0461 (6) Å	*T* = 294 K
β = 90.871 (3)°	Block, colorless
*V* = 1889.84 (13) Å^3^	0.35 × 0.20 × 0.15 mm
*Z* = 4	

### Data collection

**Table:**

Rigaku Saturn724+ diffractometer	3685 reflections with *I* > 2σ(*I*)
Radiation source: fine-focus sealed tube	*R*_int_ = 0.038
Graphite monochromator	θ_max_ = 28.7°, θ_min_ = 3.2°
ω scans	*h* = −18→20
Absorption correction: multi-scan (*CrystalClear-SM Expert*; Rigaku, 2011)	*k* = −7→5
*T*_min_ = 0.918, *T*_max_ = 0.948	*l* = −30→30
18118 measured reflections	3 standard reflections every 120 min
4737 independent reflections	intensity decay: 1%

### Refinement

**Table:**

Refinement on *F*^2^	Primary atom site location: structure-invariant direct methods
Least-squares matrix: full	Secondary atom site location: difference Fourier map
*R*[*F*^2^ > 2σ(*F*^2^)] = 0.043	Hydrogen site location: inferred from neighbouring sites
*wR*(*F*^2^) = 0.109	H atoms treated by a mixture of independent and constrained refinement
*S* = 1.08	*w* = 1/[σ^2^(*F*_o_^2^) + (0.0441*P*)^2^ + 1.2113*P*] where *P* = (*F*_o_^2^ + 2*F*_c_^2^)/3
4737 reflections	(Δ/σ)_max_ < 0.001
248 parameters	Δρ_max_ = 0.90 e Å^−^^3^
0 restraints	Δρ_min_ = −0.39 e Å^−^^3^

### Special details

**Table:**

Geometry. All esds (except the esd in the dihedral angle between two l.s. planes) are estimated using the full covariance matrix. The cell esds are taken into account individually in the estimation of esds in distances, angles and torsion angles; correlations between esds in cell parameters are only used when they are defined by crystal symmetry. An approximate (isotropic) treatment of cell esds is used for estimating esds involving l.s. planes.
Refinement. Refinement of F^2^ against ALL reflections. The weighted R-factor wR and goodness of fit S are based on F^2^, conventional R-factors R are based on F, with F set to zero for negative F^2^. The threshold expression of F^2^ > 2sigma(F^2^) is used only for calculating R-factors(gt) etc. and is not relevant to the choice of reflections for refinement. R-factors based on F^2^ are statistically about twice as large as those based on F, and R- factors based on ALL data will be even larger.

### Fractional atomic coordinates and isotropic or equivalent isotropic displacement parameters (Å^2^)

**Table:**

	*x*	*y*	*z*	*U*_iso_*/*U*_eq_	
Cl1	0.58707 (3)	0.36179 (10)	0.038690 (19)	0.03077 (13)	
Cl2	0.59584 (3)	−0.32700 (10)	0.21081 (2)	0.03171 (14)	
O1	0.45045 (7)	0.0767 (2)	0.14650 (5)	0.0203 (3)	
N1	0.37932 (9)	0.0888 (3)	0.25917 (6)	0.0175 (3)	
N2	0.36785 (9)	−0.1165 (3)	0.29187 (6)	0.0192 (3)	
C1	0.36816 (10)	−0.0354 (3)	0.15680 (7)	0.0180 (3)	
H1	0.3777	−0.2066	0.1699	0.022*	
C2	0.30805 (10)	−0.0383 (3)	0.10389 (7)	0.0173 (3)	
C3	0.24565 (11)	−0.2256 (4)	0.09721 (7)	0.0204 (4)	
H3	0.2419	−0.3505	0.1250	0.024*	
C4	0.18898 (11)	−0.2275 (4)	0.04948 (8)	0.0226 (4)	
H4	0.1473	−0.3527	0.0454	0.027*	
C5	0.19463 (11)	−0.0432 (4)	0.00800 (7)	0.0230 (4)	
H5	0.1570	−0.0448	−0.0241	0.028*	
C6	0.25637 (11)	0.1439 (4)	0.01424 (8)	0.0236 (4)	
H6	0.2600	0.2682	−0.0137	0.028*	
C7	0.31302 (11)	0.1467 (4)	0.06211 (7)	0.0214 (4)	
H7	0.3544	0.2729	0.0661	0.026*	
C8	0.32805 (11)	0.1131 (4)	0.20580 (7)	0.0208 (4)	
H8A	0.3250	0.2869	0.1948	0.025*	
H8B	0.2687	0.0551	0.2123	0.025*	
C9	0.44260 (11)	0.2432 (4)	0.27907 (8)	0.0213 (4)	
H9	0.4532 (13)	0.394 (4)	0.2614 (9)	0.026 (5)*	
C10	0.42719 (10)	−0.0863 (3)	0.33553 (7)	0.0172 (3)	
C11	0.44000 (11)	−0.2431 (4)	0.38429 (7)	0.0225 (4)	
H11	0.4076	−0.3881	0.3887	0.027*	
C12	0.50195 (12)	−0.1731 (4)	0.42472 (8)	0.0249 (4)	
H12	0.5111	−0.2719	0.4574	0.030*	
C13	0.55255 (11)	0.0458 (4)	0.41821 (8)	0.0248 (4)	
H13	0.5946	0.0856	0.4464	0.030*	
C14	0.54104 (11)	0.1992 (4)	0.37171 (8)	0.0241 (4)	
H14	0.5747	0.3422	0.3678	0.029*	
C15	0.47645 (10)	0.1351 (3)	0.32951 (7)	0.0190 (4)	
C16	0.50628 (10)	−0.0652 (4)	0.10963 (7)	0.0220 (4)	
H16A	0.4926	−0.0320	0.0691	0.026*	
H16B	0.4992	−0.2416	0.1168	0.026*	
C17	0.59832 (10)	0.0160 (4)	0.12443 (7)	0.0200 (4)	
C18	0.64021 (11)	0.2102 (4)	0.09629 (7)	0.0225 (4)	
C19	0.72309 (12)	0.2942 (4)	0.11220 (8)	0.0256 (4)	
H19	0.7488	0.4252	0.0924	0.031*	
C20	0.76702 (12)	0.1802 (4)	0.15798 (8)	0.0254 (4)	
H20	0.8229	0.2340	0.1688	0.031*	
C21	0.72823 (11)	−0.0131 (4)	0.18762 (7)	0.0242 (4)	
H21	0.7577	−0.0902	0.2183	0.029*	
C22	0.64479 (11)	−0.0904 (4)	0.17093 (7)	0.0223 (4)	

### Atomic displacement parameters (Å^2^)

**Table:**

	*U*^11^	*U*^22^	*U*^33^	*U*^12^	*U*^13^	*U*^23^
Cl1	0.0290 (2)	0.0384 (3)	0.0247 (2)	0.0036 (2)	−0.00486 (17)	0.0070 (2)
Cl2	0.0240 (2)	0.0412 (3)	0.0298 (2)	−0.0085 (2)	−0.00291 (17)	0.0100 (2)
O1	0.0146 (5)	0.0249 (7)	0.0213 (6)	−0.0006 (5)	0.0014 (4)	−0.0040 (5)
N1	0.0169 (6)	0.0201 (8)	0.0155 (6)	−0.0005 (6)	0.0000 (5)	0.0019 (6)
N2	0.0184 (7)	0.0199 (8)	0.0194 (7)	−0.0031 (6)	−0.0007 (5)	0.0021 (6)
C1	0.0146 (7)	0.0193 (10)	0.0199 (8)	−0.0005 (6)	−0.0013 (6)	0.0025 (7)
C2	0.0137 (7)	0.0183 (9)	0.0197 (8)	0.0026 (6)	−0.0004 (6)	−0.0009 (7)
C3	0.0205 (8)	0.0187 (10)	0.0219 (8)	−0.0004 (7)	0.0004 (6)	0.0021 (7)
C4	0.0195 (8)	0.0207 (10)	0.0275 (9)	−0.0021 (7)	−0.0029 (7)	−0.0043 (7)
C5	0.0192 (8)	0.0304 (11)	0.0194 (8)	0.0035 (7)	−0.0043 (6)	−0.0034 (7)
C6	0.0243 (9)	0.0246 (11)	0.0219 (8)	0.0022 (7)	−0.0023 (7)	0.0050 (7)
C7	0.0178 (8)	0.0215 (10)	0.0248 (8)	−0.0024 (7)	−0.0023 (6)	0.0031 (7)
C8	0.0160 (8)	0.0290 (11)	0.0173 (7)	0.0038 (7)	−0.0023 (6)	0.0020 (7)
C9	0.0212 (8)	0.0183 (10)	0.0243 (8)	−0.0034 (7)	0.0013 (7)	0.0027 (7)
C10	0.0159 (7)	0.0182 (9)	0.0175 (7)	0.0002 (6)	0.0004 (6)	−0.0014 (6)
C11	0.0251 (9)	0.0205 (10)	0.0218 (8)	0.0013 (7)	0.0002 (7)	0.0026 (7)
C12	0.0267 (9)	0.0268 (11)	0.0209 (8)	0.0081 (8)	−0.0027 (7)	0.0012 (7)
C13	0.0195 (8)	0.0304 (11)	0.0243 (8)	0.0051 (7)	−0.0067 (7)	−0.0070 (8)
C14	0.0189 (8)	0.0230 (11)	0.0303 (9)	−0.0029 (7)	−0.0020 (7)	−0.0051 (8)
C15	0.0166 (7)	0.0195 (10)	0.0210 (8)	−0.0008 (7)	0.0023 (6)	−0.0011 (7)
C16	0.0166 (8)	0.0303 (11)	0.0192 (8)	0.0020 (7)	−0.0005 (6)	−0.0058 (7)
C17	0.0164 (8)	0.0269 (11)	0.0167 (7)	0.0030 (7)	0.0011 (6)	−0.0063 (7)
C18	0.0212 (8)	0.0300 (11)	0.0162 (7)	0.0047 (7)	−0.0002 (6)	−0.0012 (7)
C19	0.0241 (9)	0.0298 (12)	0.0230 (8)	−0.0021 (8)	0.0028 (7)	0.0028 (8)
C20	0.0194 (8)	0.0341 (12)	0.0226 (8)	−0.0024 (8)	−0.0020 (7)	−0.0010 (8)
C21	0.0194 (8)	0.0336 (12)	0.0195 (8)	0.0005 (7)	−0.0035 (6)	0.0011 (8)
C22	0.0186 (8)	0.0297 (11)	0.0188 (8)	0.0005 (7)	0.0007 (6)	−0.0009 (7)

### Geometric parameters (Å, º)

**Table:**

Cl1—C18	1.7467 (18)	C9—H9	0.92 (2)
Cl2—C22	1.7446 (19)	C10—C11	1.417 (2)
O1—C1	1.4150 (19)	C11—C12	1.369 (2)
O1—C16	1.432 (2)	C11—H11	0.9300
N1—N2	1.350 (2)	C12—H12	0.9300
N1—C8	1.453 (2)	C13—C12	1.417 (3)
N1—C9	1.348 (2)	C13—C14	1.362 (3)
N2—C10	1.352 (2)	C13—H13	0.9300
C1—C2	1.514 (2)	C14—H14	0.9300
C1—C8	1.519 (2)	C15—C9	1.392 (2)
C1—H1	0.9800	C15—C10	1.416 (2)
C2—C7	1.388 (2)	C15—C14	1.416 (2)
C3—C2	1.393 (2)	C16—H16A	0.9700
C3—C4	1.388 (2)	C16—H16B	0.9700
C3—H3	0.9300	C17—C16	1.503 (2)
C4—H4	0.9300	C17—C18	1.390 (3)
C5—C4	1.381 (3)	C17—C22	1.398 (2)
C5—C6	1.384 (3)	C18—C19	1.386 (3)
C5—H5	0.9300	C19—H19	0.9300
C6—C7	1.391 (2)	C20—C19	1.384 (3)
C6—H6	0.9300	C20—H20	0.9300
C7—H7	0.9300	C21—C20	1.382 (3)
C8—H8A	0.9700	C21—C22	1.387 (2)
C8—H8B	0.9700	C21—H21	0.9300
			
C1—O1—C16	114.10 (14)	C15—C10—C11	120.81 (16)
N2—N1—C8	118.27 (14)	C10—C11—H11	121.3
C9—N1—N2	114.35 (14)	C12—C11—C10	117.45 (18)
C9—N1—C8	127.25 (15)	C12—C11—H11	121.3
N1—N2—C10	103.07 (14)	C11—C12—C13	121.84 (17)
O1—C1—C2	113.34 (13)	C11—C12—H12	119.1
O1—C1—C8	105.53 (14)	C13—C12—H12	119.1
O1—C1—H1	108.9	C12—C13—H13	119.2
C2—C1—C8	111.02 (13)	C14—C13—C12	121.56 (17)
C2—C1—H1	108.9	C14—C13—H13	119.2
C8—C1—H1	108.9	C13—C14—C15	118.20 (18)
C3—C2—C1	120.00 (15)	C13—C14—H14	120.9
C7—C2—C1	120.84 (15)	C15—C14—H14	120.9
C7—C2—C3	119.15 (15)	C9—C15—C10	104.07 (15)
C2—C3—H3	119.7	C9—C15—C14	135.79 (18)
C4—C3—C2	120.55 (17)	C14—C15—C10	120.12 (16)
C4—C3—H3	119.7	O1—C16—C17	105.74 (14)
C3—C4—H4	120.0	O1—C16—H16A	110.6
C5—C4—C3	119.91 (17)	O1—C16—H16B	110.6
C5—C4—H4	120.0	C17—C16—H16A	110.6
C4—C5—C6	120.00 (16)	C17—C16—H16B	110.6
C4—C5—H5	120.0	H16A—C16—H16B	108.7
C6—C5—H5	120.0	C18—C17—C16	123.10 (16)
C5—C6—C7	120.22 (17)	C18—C17—C22	115.75 (15)
C5—C6—H6	119.9	C22—C17—C16	120.99 (17)
C7—C6—H6	119.9	C17—C18—Cl1	119.65 (13)
C2—C7—C6	120.18 (17)	C19—C18—Cl1	117.32 (15)
C2—C7—H7	119.9	C19—C18—C17	123.03 (16)
C6—C7—H7	119.9	C18—C19—H19	120.5
N1—C8—C1	111.38 (13)	C20—C19—C18	119.05 (18)
N1—C8—H8A	109.4	C20—C19—H19	120.5
N1—C8—H8B	109.4	C19—C20—H20	119.8
C1—C8—H8A	109.4	C21—C20—C19	120.30 (17)
C1—C8—H8B	109.4	C21—C20—H20	119.8
H8A—C8—H8B	108.0	C20—C21—C22	119.08 (17)
N1—C9—C15	106.30 (16)	C20—C21—H21	120.5
N1—C9—H9	121.4 (13)	C22—C21—H21	120.5
C15—C9—H9	132.1 (13)	C17—C22—Cl2	119.14 (13)
N2—C10—C11	126.94 (17)	C21—C22—Cl2	118.07 (14)
N2—C10—C15	112.20 (15)	C21—C22—C17	122.78 (18)
			
C16—O1—C1—C2	−69.95 (19)	C10—C11—C12—C13	−0.8 (3)
C16—O1—C1—C8	168.36 (13)	C14—C13—C12—C11	1.0 (3)
C1—O1—C16—C17	−155.79 (14)	C12—C13—C14—C15	0.2 (3)
C8—N1—N2—C10	177.04 (14)	C10—C15—C9—N1	−0.20 (18)
C9—N1—N2—C10	0.86 (18)	C14—C15—C9—N1	−178.25 (19)
N2—N1—C8—C1	−80.77 (18)	C9—C15—C10—N2	0.76 (19)
C9—N1—C8—C1	94.9 (2)	C9—C15—C10—C11	−176.80 (16)
N2—N1—C9—C15	−0.4 (2)	C14—C15—C10—N2	179.19 (15)
C8—N1—C9—C15	−176.19 (15)	C14—C15—C10—C11	1.6 (2)
N1—N2—C10—C11	176.40 (16)	C9—C15—C14—C13	176.39 (19)
N1—N2—C10—C15	−0.98 (18)	C10—C15—C14—C13	−1.4 (3)
O1—C1—C2—C3	151.29 (16)	C18—C17—C16—O1	−90.65 (19)
O1—C1—C2—C7	−29.9 (2)	C22—C17—C16—O1	84.6 (2)
C8—C1—C2—C3	−90.1 (2)	C16—C17—C18—Cl1	−3.0 (2)
C8—C1—C2—C7	88.65 (19)	C16—C17—C18—C19	176.14 (17)
O1—C1—C8—N1	−64.67 (18)	C22—C17—C18—Cl1	−178.45 (13)
C2—C1—C8—N1	172.15 (15)	C22—C17—C18—C19	0.7 (3)
C1—C2—C7—C6	−178.90 (16)	C16—C17—C22—Cl2	1.9 (2)
C3—C2—C7—C6	−0.1 (3)	C16—C17—C22—C21	−177.02 (17)
C4—C3—C2—C1	178.70 (15)	C18—C17—C22—Cl2	177.45 (13)
C4—C3—C2—C7	−0.1 (3)	C18—C17—C22—C21	−1.4 (3)
C2—C3—C4—C5	0.4 (3)	Cl1—C18—C19—C20	179.44 (15)
C6—C5—C4—C3	−0.4 (3)	C17—C18—C19—C20	0.3 (3)
C4—C5—C6—C7	0.2 (3)	C21—C20—C19—C18	−0.6 (3)
C5—C6—C7—C2	0.1 (3)	C22—C21—C20—C19	−0.2 (3)
N2—C10—C11—C12	−177.68 (17)	C20—C21—C22—Cl2	−177.68 (15)
C15—C10—C11—C12	−0.5 (2)	C20—C21—C22—C17	1.2 (3)

### Hydrogen-bond geometry (Å, º)

Cg2 and Cg3 are the centroids of the C2–C7 and C10–C15 rings, respectively.

**Table:**

*D*—H···*A*	*D*—H	H···*A*	*D*···*A*	*D*—H···*A*
C4—H4···*Cg*3^i^	0.93	2.91	3.591 (2)	131
C11—H11···*Cg*2^i^	0.93	2.87	3.616 (2)	138

Symmetry code: (i) −*x*+1/2, *y*−1/2, −*z*+1/2.
